# Variations in *TM6SF2*, *PCSK9* and *PCSK7* genes and risk of hepatic steatosis after liver transplantation: a cross-sectional study

**DOI:** 10.1186/s12876-021-02041-8

**Published:** 2021-12-07

**Authors:** Ahad Eshraghian, Elham Moasser, Negar Azarpira, Mohammad Reza Fattahi, Saman Nikeghbalian, Seyed Ali Malek-Hosseini, Bita Geramizadeh

**Affiliations:** 1 Shiraz Transplant Center, Abu-Ali Sina Hospital, Shiraz University of Medical Sciences, Shiraz, Iran; 2 Transplant Research Center, Shiraz University of Medical Sciences, Research Tower, PO Box 71994-67985, Shiraz, Iran; 3 Gastroenterohepatology Research Center, Shiraz University of Medical Sciences, Shiraz, Iran

**Keywords:** Liver transplant, Hepatic steatosis, Non-alcoholic fatty liver disease, Hepatic steatosis, Genetic variants

## Abstract

**Background:**

Genetic abnormalities might have important role in pathogenesis of hepatic steatosis after liver transplantation. We aimed to investigate association between genetic variations in transmembrane 6 superfamily member 2 (*TM6SF2)* rs58542926, proprotein convertase subtilisin/kexin type 9 (*PCSK9)* rs505151 and proprotein convertase subtilisin/kexin type 7 (*PCSK7*) rs2277287 with hepatic steatosis in liver transplant recipients.

**Methods:**

In a cross-sectional study, adult (> 18 years) liver transplant recipients who were referred for their routine post-transplant follow-up between June 2018 and September 2018 were included in the study. Hepatic steatosis in transplant recipients was assessed by controlled attenuation parameter (CAP). Polymerase chain reaction-restriction fragment length polymorphism (PCR–RFLP) was used to study *TM6SF2* rs58542926, *PCSK7* rs2277287 and *PCSK9* rs505151 genotypes.

**Results:**

107 liver transplant recipients were included. There was no association between different genotypes of *PCSK9* rs505151 and *PCSK7* rs2277287 with hepatic steatosis in liver transplant recipients (*P* value > 0.05). The presence of TT genotype of *TM6SF2* rs58542926 was higher in patients with hepatic steatosis measured by CAP after liver transplantation. In patients with moderate and severe hepatic steatosis (grade 2 and 3 steatosis), AG + GG genotypes of PCSK9 rs505151 were more prevalent than AA genotype (OR 8.667; 95% CI 1.841–40.879; *P* value = 0.004) compared to patients with mild steatosis (grade 1). In multivariate regression model, AG + GG genotypes of *PCSK9* rs505151 were associated with moderate and severe steatosis in liver transplant recipients (OR 5.747; 95% CI 1.086–30.303; *P* value = 0.040).

**Conclusions:**

Genetic variations in *TM6SF2* rs58542926 and *PCSK9* rs505151 might be associated with hepatic steatosis in liver transplant recipients.

## Introduction

Clinical outcomes of liver transplant recipients have been improved during recent years leading to increased survival of patients. In parallel, chronic adverse outcomes including metabolic abnormalities have been increased [1, 2]. Hepatic steatosis might occur after liver transplantation with an estimated reported prevalence of 30–60% in different studies [3, 4]. Pathogenesis of hepatic steatosis after liver transplantation has not been well elucidated yet. We have recently demonstrated that alterations in serum adipokines and insulin resistance are main contributing factors in hepatic steatosis after liver transplantation [5]. Genetic susceptibility might also have important role in pathogenesis of non-alcoholic fatty liver disease (NAFLD) [6]. Patatin like phospholipase domain-containing 3 (PNPLA3) rs738409 polymorphism has been reported to be associated with hepatic steatosis, steatohepatitis and liver fibrosis in patients with NAFLD [7, 8]. Similarly, genetic predisposition might be a risk factor for hepatic steatosis in liver transplant recipients. It has been reported that liver transplant recipients with G allele in position rs738409 of PNPLA3 were at increased risk for graft steatosis [9]. Transmembrane 6 superfamily member 2 (*TM6SF2*) is a gene encoding a transmembrane protein located in endoplasmic reticulum and Golgi apparatus and acts as a lipid transporter [10]. *TM6SF2* rs58542926 variant was shown to be associated with hepatic steatosis independent of other metabolic risk factors [11]. It has been suggested that *TM6SF2* rs58542926 variant is associated with hepatic steatosis via affecting glucose homeostasis, nutrient oxidation, postprandial lipoprotein and adipokine [12]. However, its impact on graft steatosis after liver transplantation is not clear. Proprotein convertase subtilisin/kexin type 9 (*PCSK9*) is an endoprotease synthesized in liver and is involved in clearance of low density lipoprotein (LDL) in hepatocytes [13]. Two recent studies have shown circulating *PCSK9* was associated with hepatic steatosis [14] and *PCSK9* rs11591147 loss of function mutation had protective effect against hepatic steatosis [15]. Proprotein convertase subtilisin/kexin type 7 (*PCSK7*) is another endoprotease from subtilisin-like proprotein convertase family that is involved in lipid metabolism [16]. Although, *PCSK9* and *PCSK7* can theoretically be associated with hepatic steatosis there is few clinical studies in this regard. We conducted this study to explore the association between genetic variations in *TM6SF2* rs58542926, *PCSK9* rs505151 and *PCSK7* rs2277287 and hepatic steatosis in liver transplant recipients.

## Methods

In a cross-sectional study, liver transplant recipients who had undergone liver transplantation from deceased donors at Shiraz Transplant Center, Shiraz, Iran, were included. Patients were referred for their routine post-transplant follow-up between June 2018 and September 2018. Patients were included if: (a) they were adult (> 18 years), (b) at least 6 months had been passed from transplant surgery, (c) and did not have malignancy. We used tacrolimus based immunosuppressive regimen for all patients as maintenance of immunosuppression after liver transplantation. Patients with acute rejection episodes and those with serum aspartate aminotransferase and/or alanine aminotransferase levels higher than 5 times upper limit of normal range were excluded from the study. Clinical characteristics of patients including age, gender, underlying liver disease, post-transplant diabetes (PTDM), hyperlipidemia, hypertension and laboratory data were recorded at the time of study inclusion. Body mass index (BMI) was calculated using this formula: weight (kg) divided by height (m^2^) squared. Hepatic steatosis in transplant recipients was assessed by controlled attenuation parameter (CAP).

We used CAP measurement during vibration controlled transient elastography (VCTE) (FibroScan^®^, Echosens, Paris, France) for estimation of hepatic steatosis in all liver transplant recipients. CAP measurement was done after an overnight fasting on the day of clinical visit and blood sampling. All CAP measurements were performed by one person with sufficient expertise with the procedure. CAP measurement was considered to be reliable if 10 valid successful acquisitions were obtained. CAP measurement was performed using M probe in all patients. In obese patients, in whom use of M probe was failed, XL probe was used for CAP measurement. CAP measurement was expressed in decibel per meter (dB/m) [17]. The cutoff scores for CAP estimation of hepatic steatosis were: ≥ 238 dB/m and < 259 dB/m for S1 (mild steaosis) (corresponding to 11–32% liver fat), ≥ 259 dB/m and < 292 dB/m for S2 (moderate steatosis) (33–65% liver fat) and ≥ 292 dB/m for S3 (severe steatosis) (≥ 66% liver fat) [17].

### Genotyping

Whole blood was collected from the liver transplant recipients in EDTA tubes and the genomic DNA was extracted from the peripheral blood leukocytes using high yield DNA purification Sinaclon Kit DNP™ protocol (SINACLON, Tehran, Iran) according to the manufacturer’s instructions. The DNA extracted kit was placed in room temperature and all phases of DNA extraction were done under laminar hood. All instruments such as samplers and sampler tips, micro tubes, racks were sterilized by autoclave and 70% alcohol. Blood tubes were placed in room temperature. DNA was stored at − 20 °C until the time of usage.

The polymerase chain reaction (PCR) conditions for determining *TM6SF2*, *PCSK9*, and *PCSK7* genotypes were set up. Briefly, the PCR protocol was performed using 300–500 ng of genomic DNA in total volume of 25 µL, 200 µM dNTPs, 10 pmol of each primer, 1.5 mM MgCl2, and 1 U Taq polymerase enzyme in a 10 mM PCR buffer. The PCR protocol consisted of 5 min at 95 °C, 35 cycles of 1 min at 95 °C, 1 min at 60 °C, 45 s at 72 °C, and then 5 min at 72 °C. Electrophoresis of 10 µL of PCR products was performed on a 1.5% agarose gel as described before [18, 19]. Negative controls (tubes containing the PCR mixture, without the DNA template) were incubated in every run for prevention of contamination.

Polymerase chain reaction-restriction fragment length polymorphism (PCR–RFLP) was used to study *TM6SF2* rs58542926, *PCSK7* rs2277287 and *PCSK9* rs505151 genotypes. To determine the *TM6SF2* genotypes, the PCR product was digested using Hpy188I (New England Biolabs (NEB), UK) restriction enzyme at 37 °C for 16 h. The rs2277287 polymorphism in the *PCSK7* gene was studied using restriction endonuclease PvuII (New England Biolabs (NEB), UK) and the *PCSK9*-rs505151 variation was studied using Eam1104I (EarI) restriction enzyme (Thermo Fisher scientific, Inc, Waltham, MA, USA) (Table 1). The digested fragments were electrophoresed in 3% agarose gel and visualized with ultraviolet illumination. Furthermore, 10% of the PCR product underwent direct sequencing to verify the result of gel electrophoresis. There was complete agreement between the two methods.

**Table 1:** Primers used for TM6SF2, PCSK7, and PCSK9 polymorphisms typing

SNP	Primer (5′ → 3′)	Annealing temperature (°C)	Amplicon (bp)	Restriction enzyme	RFLP fragments (bp)
*TM6SF2*-rs58542926	F: CCAGGTGTTGTCTCAAGTGG R: CCAGGTGTTGTCTCAAGTGG	60	247	*Hpy188I*	T allele: 247 C allele: 175 + 71
*PCSK7*-rs2277287	F: CATTGCCTAGGTATCCGGGT R: GGGCTTCTCATGTGGCAATC	60	321	*PvuII*	T allele: 321 C allele: 226 + 95
*PCSK9*-rs505151	F: CACGGTTGTGTCCCAAATGG R: GAGAGGGACAAGTCGGAACC	60	440	*Eam1104I (EarI)*	G allele: 440 A allele: 290 + 150

### Statistical analysis

Student’s t-test, Mann–Whitney U test and Chi-square test were used to analyze continuous and categorical variables. Data were presented using means ± standard deviation for numeric variables, and percentages/counts for categorical variables. Logistic regression analysis was used to identify the independent variables and association of genetic variations with hepatic steatosis after liver transplantation. Statistically significant variables in univariate analysis were included in multivariate analysis. A *P* value of < 0.05 was considered statistically significant. Statistical analysis was performed with SPSS 20.0 (SPSS Inc.; Chicago, IL, USA).

## Results

We evaluated 107 liver transplant recipients who had undergone liver transplantation for different etiologies of liver cirrhosis. Baseline characteristics of patients are outlined in Table 2. Mean time from transplant surgery to measurement of CAP was 41.16 ± 35.85 months. Thirty seven patients (34.6%) were diagnosed to have different grades of hepatic steatosis in TE with a mean CAP of 207.76 ± 56.84 (dB/m). Seventeen patients (45.9%) had grade 1 hepatic steatosis, 11 patients (29.7%) had grade 2 hepatic steatosis and 9 patients (24.3%) had grade 3 hepatic steatosis during CAP measurement.

**Table 2:** Baseline characteristics of patients in the study

Age (years)	43.21 ± 12.99
Sex (female/male), n (%)	47/60 (43.9/56.1)
BMI (kg/m^2^)	24.92 ± 5.17
PTDM, n (%)	33 (32.4)
HTN, n (%)	26 (26)
HLP, n (%)	27 (26.5)
Rejection, n (%)	31 (35.2)
Etiology of liver disease (n, %)	
HBV	19 (17.7)
HCV	4 (3.7)
NASH	14 (13)
AIH	11 (10.2)
PSC	21 (19.6)
Cryptogenic	15 (14)
Others	23 (21.4)
AST (IU/L)	19 (9–164)
ALT (IU/L)	22.5 (8–226)
Alk.Phos (IU/L)	200 (44–800)
Steatosis in TE (n, %)	37 (34.6)
Mean CAP (dB/m)	207.76 ± 56.84
TM6SF2 rs58542926 genotype (n, %)	
CC	77 (71.9)
CT	28 (26.1)
TT	2 (1.8)
PCSK9 rs505151 genotype (n, %)	
AA	54 (50.4)
AG	50 (46.7)
GG	3 (2.8)
PCSK7 rs2277287 genotype (n, %)	
CC	26 (24.2)
CT	44 (41.1)
TT	37 (34.5)

*BMI* body mass index, *PTDM* post transplant diabetes mellitus, *HLP* hyperlipidemia, *HTN* hypertension, *HBV* hepatitis B virus, *HCV* hepatitis C virus, *NASH* non-alcoholic steatohepatitis, *AIH* autoimmune hepatitis, *PSC* primary sclerosing cholangitis, *AST* aspartate aminotransferase, *ALT* alanine aminotransferase, *Alk.Phos* alkaline phosphatase, *TE* transient elastography, *TM6SF2* transmembrane 6 super family 2, *PCSK9* proprotein convertase subtilisin/kexin type 9, *PCSK7* proprotein convertase subtilisin/kexin type 7

**Fig. 1:**
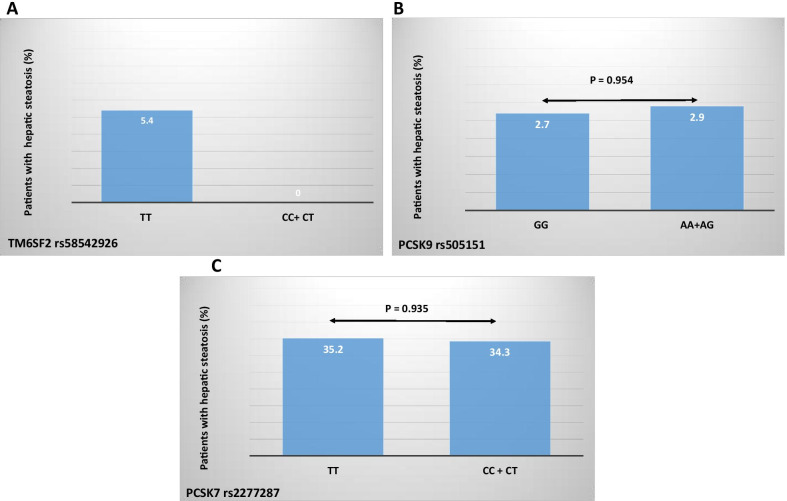
Genetic variants of TM6SF2, PCSK9 and PCSK7 genes in liver transplant recipients with and without hepatic steatosis

The presence of steatosis in liver allografts after liver transplantation based on different variants of *TM6SF2* rs58542926, PCSK9 rs505151 and *PCSK7* rs2277287 genes are shown in Fig. 1. There was no association between different genotypes of *PCSK9* rs505151 and *PCSK7* rs2277287 with hepatic steatosis in liver transplant recipients (*P* value > 0.05). The TT genotype of *TM6SF2* rs58542926 was occurred in 5.4% of patients with hepatic steatosis and no patients without steaosis measured by CAP after liver transplantation. Univariate and multivariate analysis of risk factors for presence of hepatic steatosis after liver transplantation are outlined in Table 3. Higher age and BMI, post-transplant diabetes mellitus, post-transplant hyperlipidemia and hypertension were associated with hepatic steatosis in univariate analysis. In multivariate regression model, none of these variables were independent predictors of hepatic steatosis after liver transplantation among our study population (Table 3).

**Table 3:** Comparison of genetic variants and other risk factors in patients with and without hepatic steatosis after liver transplantation

Univariate				Multivariate analysis		
	Steatosis (+)	Steatosis (−)	*P* value	OR	95% CI	*P* value
Age (year)	49.83 ± 10.66	39.71 ± 12.81	< 0.001	1.144	0.904–1.448	0.253
Sex (male/female)	23/14	37/33	0.356			
Height (cm)	168.16 ± 10.47	166.73 ± 9.36	0.476			
Weight (kg)	80.83 ± 17.42	63.82 ± 11.58	< 0.001			
BMI (kg/m^2^)	28.59 ± 5.70	22.92 ± 3.54	< 0.001	1.074	0.768–1.503	0.675
HC (cm)	111.83 ± 11.50	97.29 ± 8.14	< 0.001	1.013	0.732–1.402	0.938
WC (cm)	104.49 ± 12.04	88.17 ± 9.29	< 0.001	1.114	0.827–1.501	0.476
AST (IU/L)	22.19 ± 12	26.41 ± 25.71	0.397			
ALT (IU/L)	24.76 ± 13.35	32.14 ± 34.70	0.267			
Alk.Phos (IU/L)	245.46 ± 105.77	212.35 ± 185.23	0.370			
PTDM	61.1%	16.7%	< 0.001	0.168	0.008–3.647	0.256
Post-transplant HLP	47.2%	15.2%	< 0.001	1.222	0.085–17.64	0.883
Post-transplant HTN	38.9%	18.8%	0.028	0.324	0.017–6.318	0.457
Rejection	36.7%	34.5%	0.839			
Mean prednisolone dose (mg)	8.26 ± 3.87	7.20 ± 3.74	0.379			
TM6SF2 genotype				1.237	0.083–18.473	0.878
TT versus CC + CT	5.4%	0				
PCSK9 genotype						
GG versus AA + AG	2.7%	2.9%	0.954			
PCSK7 genotype						
TT versus CC + CT	35.2%	34.3%	0.935			

*BMI* body mass index, *AST* aspartate aminotransferase, *ALT* alanine aminotransferase, *Alk.Phos* alkaline phosphatase, *PTDM* post-transplant diabetes mellitus, *HLP* hyperlipidemia, *HTN* hypertension, *WC* waist circumference, *HC* hip circumference, *TM6SF2* transmembrane 6 super family 2, *PCSK9* proprotein convertase subtilisin/kexin type 9, *PCSK7* proprotein convertase subtilisin/kexin type 7

**Fig. 2:**
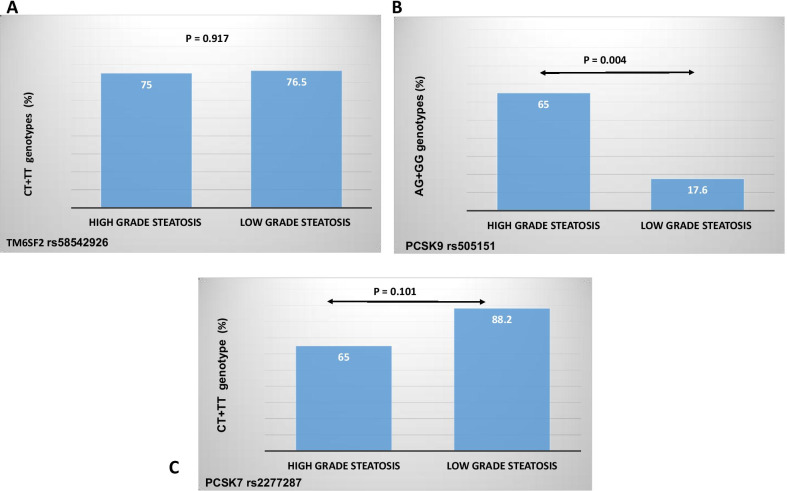
Genetic variants of TM6SF2, PCSK9 and PCSK7 genes in liver transplant recipients with high grade (moderate to severe) steatosis and low grade (mild) steatosis

Patients were divided into those with mild steatosis (grade 1 steatosis) and those with moderate to severe steatosis (grade 2 and 3 steatosis). There were 17 patients (45.9%) with mild steatosis (grade 1) and 20 patients (54%) with moderate to severe steatosis (grade 2 and 3 steatosis). The presence of mild, moderate and severe steatosis in liver allografts based on different variants of *TM6SF2* rs58542926, *PCSK9* rs505151 and *PCSK7* rs2277287 genes are shown in Fig. 1. There was no association between different genotypes of *TM6SF2* rs58542926 and *PCSK7* rs2277287 with moderate to severe hepatic steatosis in liver transplant recipients (*P* value > 0.05). In patients with moderate to severe hepatic steatosis, AG + GG genotypes of *PCSK9* rs505151 were more prevalent compared to AA genotype (OR 8.667; 95% CI 1.841–40.879; *P* value = 0.004) (Fig. 2). Other risk factors for presence of moderate to severe versus mild hepatic steatosis after liver transplantation are outlined in Table 4. In multivariate regression model, AG + GG genotypes of *PCSK9* rs505151 were associated with moderate to severe hepatic steatosis in liver transplant recipients (OR 5.747; 95% CI 1.086–30.303; *P* value = 0.040).

**Table 4:** Comparison of genetic variants and other risk factors in patients with different grades of hepatic steatosis after liver transplantation

Univariate				Multivariate analysis		
	Moderate to severe steatosis	Mild steatosis	*P* value	OR	95% CI	*P* value
Age (year)	51.90 ± 9.94	47.41 ± 11.27	0.207			
Sex (male/female)	10/10	13/4	0.098			
Height (cm)	167.10 ± 11.55	169.41 ± 9.23	0.511			
Weight (kg)	86 ± 16.89	74.76 ± 16.47	0.049			
BMI (kg/m^2^)	30.81 ± 5.11	25.99 ± 5.38	0.008	1.055	0.855–1.301	0.620
WC (cm)	109.74 ± 11.74	99.29 ± 9.95	0.006	1.062	0.953–1.183	0.276
AST (IU/L)	21.62 ± 9.4	23.92 ± 13.61	0.590			
ALT (IU/L)	24.43 ± 11.76	26.57 ± 13.74	0.650			
PTDM	57.9%	64.7%	0.676			
Post-transplant HLP	47.4%	47.1%	0.985			
Post-transplant HTN	42.1%	35.3%	0.676			
Rejection	36.7%	34.5%	0.839			
Mean prednisolone dose (mg)	8.26 ± 3.87	7.20 ± 3.74	0.379			
TM6SF2 genotype						
CT + TT versus CC	75%	76.5%	0.917			
PCSK9 genotype						
AG + GG versus AA	65%	17.6%	0.004	5.747	1.086–30.303	0.040
PCSK7 genotype						
CT + TT versus CC	65%	88.2%	0.101			

*BMI* body mass index, *AST* aspartate aminotransferase, *ALT* alanine aminotransferase, *PTDM* post-transplant diabetes mellitus, *HLP* hyperlipidemia, *HTN* hypertension, *WC* waist circumference, *HC* hip circumference, *TM6SF2* transmembrane 6 super family 2, *PCSK9* proprotein convertase subtilisin/kexin type 9, *PCSK7* proprotein convertase subtilisin/kexin type 7

## Discussion

In this study, we investigated the association between variations in 3 candidate genes and hepatic steatosis after liver transplantation. Our results showed that frequency of TT genotype of *TM6SF2* rs58542926 was higher in patients with hepatic steatosis than in patients without hepatic steatosis. In subgroup analysis, comparing patients with mild steatosis to patients with moderate to severe steatosis, AG + GG genotypes of *PCSK9* rs505151 were associated with moderate and high grade steatosis in liver transplant recipients. Variations in *PCSK7* rs2277287 gene was associated neither with presence nor degree of hepatic steatosis in liver transplant recipients.

*TM6SF2* rs58542926 T allele and TT genotype are well known genetic risk factors for NAFLD, non-alcoholic steatohepatitis (NASH) and liver cirrhosis [20, 21]. This polymorphism changes glutamic acid to lysine amino acid causing down-regulation of *TM6SF2* protein and increased deposition of triglycerides in hepatocytes [22]. On the other hand, overexpression of *TM6SF2* protein causes decreased number and size of fat droplets in hepatocytes [23]. Previously, Mikova et al. showed that donor rs58542926 polymorphism in *TM6SF2* gene was independently associated with graft steatosis and had additive effects on donor PNPLA3 [24]. However, a cross sectional study, with limited number of patients, failed to demonstrate association between genetic variations in recipient *TM6SF2* rs58542926 and hepatic steatosis after liver transplantation [25]. In our findings, frequency of *TM6SF2* rs58542926 TT genotype was higher in patients with hepatic steatosis after liver transplantation.

*PCSK7* is a calcium-dependent serine endopeptidase located on chromosome 11 and is involved in the process of adipogenesis [16]. Ploso et al. showed that *PCSK7* rs142953140 single nucleotide polymorphism was associated with serum lipid profile levels [26]. A genome wide association study (GWAS) showed that *PCSK7* rs508487 single nucleotide polymorphism was associated with serum total cholesterol levels [27]. *PCSK7* rs2277287 polymorphism has been recently reported to be associated with alterations in serum lipid profiles [28]. Dongiovanni et al. reported that *PCSK7* rs236918 gene variations were associated with altered lipid profile and severe liver damage [29]. The role of *PCSK7* rs2277287 gene in hepatic steatosis has not been previously investigated.

*PCSK9* is synthesized and secreted by hepatocytes and has inhibitory effects on uptake of low density lipoproteins [30]. In clinical setting, *PCSK9* rs505151 variants were associated with elevated LDL cholesterol in American Indians [13]. In a population of European ancestry, *PCSK9* rs505151 polymorphism was reported in patients with hypercholesterolemia [31]. In a meta-analysis, increased triglycerides and LDL–cholesterol were associated with G allele of *PCSK9* rs505151 [32]. It has been reported that circulating *PCSK9* was associated with severity of hepatic steatosis, ballooning and fibrosis stage [33]. Protective effects of *PCSK9* inhibitions against NAFLD and insulin resistance have been reported in recent studies [34]. Cariou et al. reported that plasma *PCSK9* concentration was correlated with hepatic steatosis and hepatic insulin resistance in a population of young healthy volunteers fed with high fructose diet [35]. In our study, liver transplant recipients with AG + GG variants of *PCSK9* rs505151 conferred an increased risk for higher degrees of hepatic steatosis as assessed by CAP.

Hepatic steatosis is an increasingly encountered problem after liver transplantation, however, pathogenesis is not clear. *PNPLA3* rs738409 polymorphism is the most studied genetic variant for steatosis after liver transplantation and has been previously reported as a risk factor [36]. In this study, we tested liver transplant recipients with hepatic steatosis for genetic polymorphisms that had not been previously investigated. Our results revealed that *TM6SF2* rs58542926 and *PCSK9* rs505151 variants might be associated with hepatic steatosis after liver transplantation. Although our study reported novel clinical observations, it is a single center experience with limited number of patients. These findings should be replicated in future larger studies before any robust conclusion. On the other hand, genetic variations in donors might have impact on hepatic steatosis after liver transplantation and testing donors for specific genetic polymorphisms can be considered in future studies. Although the clinical importance of hepatic steatosis after liver transplantation is not still clear, it has been reported that advanced graft fibrosis was more prevalent in liver transplant recipients with graft steatosis after liver transplantation [37]. Therefore, liver transplant recipients with high risk genotypes might be considered for more intense follow-up and interventions for treatment.

## Data Availability

The datasets generated and/or analysed during the current study are not publicly available due to keeping privacy of patients but are available from the corresponding author on reasonable request.
